# [Expression of Concern] Apoptotic effect of gambogic acid in esophageal squamous cell carcinoma cells via suppression of the NF-κB pathway

**DOI:** 10.3892/ol.2026.15586

**Published:** 2026-04-14

**Authors:** Wen-Yue Liu, Xu Wu, Cheng-Quan Liao, Jie Shen, Jun Li

Oncol Lett 11: 3681–3685, 2016; DOI: 10.3892/ol.2016.4437

Following the publication of the above paper, it has been drawn to the Editor's attention by a concerned reader that three pairs of data panels showing TUNEL apoptotic assay data in Fig. 2B on p. 3683 contained overlapping sections, such that images which were intended to show the results from differently performed experiments had apparently been derived from a smaller number of original sources. In addition, western blot data shown in Fig. 4 on p. 3684 were strikingly similar to data that were subsequently submitted to the journals *Molecular Medicine Reports* and *Oncology Letters* in articles written by different authors at different research institutes.

The authors have been contacted by the Editorial Office to offer an explanation for these apparent anomalies in the presentation of the data in this paper, and we are awaiting their response. Owing to the fact that the Editorial Office has been made aware of potential issues surrounding the scientific integrity of this paper, we are issuing an Expression of Concern to notify readers of these potential problems while the Editorial Office continues to investigate this matter further.

